# Hybrid PET-MRI for early detection of dopaminergic dysfunction and microstructural degradation involved in Parkinson’s disease

**DOI:** 10.1038/s42003-021-02705-x

**Published:** 2021-10-07

**Authors:** Song’an Shang, Daixin Li, Youyong Tian, Rushuai Li, Hongdong Zhao, Liyun Zheng, Yingdong Zhang, Yu-Chen Chen, Xindao Yin

**Affiliations:** 1 Department of Radiology, Nanjing First Hospital, Nanjing Medical University, Nanjing, China; 2 Department of Radiology, Nanjing Second Hospital, Nanjing University of Chinese Medicine, Nanjing, China; 3 Department of Neurology, Nanjing First Hospital, Nanjing Medical University, Nanjing, China; 4 Department of Nuclear Medicine, Nanjing First Hospital, Nanjing Medical University, Nanjing, China; 5 Central Research Institute, United Imaging Healthcare, Shanghai, China

**Keywords:** Parkinson's disease, Parkinson's disease

## Abstract

Dopamine depletion and microstructural degradation underlie the neurodegenerative processes in Parkinson’s disease (PD). To explore early alterations and underlying associations of dopamine and microstructure in PD patients utilizing the hybrid positron emission tomography (PET)-magnetic resonance imaging (MRI). Twenty-five PD patients in early stages and twenty-four matched healthy controls underwent hybrid ^18^F-fluorodopa (DOPA) PET-diffusion tensor imaging (DTI) scanning. The striatal standardized uptake value ratio (SUVR), DTI maps (fractional anisotropy, FA; mean diffusivity, MD) in subcortical grey matter, and deterministic tractography of the nigrostriatal pathway were processed. Values in more affected (MA) side, less affected (LA) side and mean were analysed. Correlations and mediations among PET, DTI and clinical characteristics were further analysed. PD groups exhibited asymmetric pattern of dopaminergic dysfunction in putamen, impaired integrity in the microstructures (nigral FA, putaminal MD, and FA of nigrostriatal projection). On MA side, significant associations between DTI metrics (nigral FA, putaminal MD, and FA of nigrostriatal projection) and motor performance were significantly mediated by putaminal SUVR, respectively. Early asymmetric disruptions in putaminal dopamine concentrations and nigrostriatal pathway microstructure were detected using hybrid PET-MRI. The findings further implied that molecular degeneration mediates the modulation of microstructural disorganization on motor dysfunction in the early stages of PD.

## Abstract (web summary)

To explore early alterations and underlying associations of dopamine levels and microstructure in Parkinson’s Disease (PD), Shang et al use a hybrid positron emission tomography (PET)-magnetic resonance imaging (MRI) approach in early stage patients and age-matched controls. Their data implies that molecular degeneration mediates the effects of microstructural disorganization on motor dysfunction in the early stages of PD.

## Introduction

Asymmetrical loss of dopaminergic neurons in the substantia nigra (SN) is the core pathological feature of Parkinson’s disease (PD), resulting in concomitantly decreased dopamine concentrations in the striatum^1^. This dopaminergic denervation is presumed to cause dysfunction in the nigrostriatal pathway and impairments in the basal ganglia circuitry, which would ultimately lead to characteristic motor symptoms (bradykinesia, resting tremor, rigidity) with unilateral onset and persistent asymmetry. Given that PD diagnosis based on clinical symptoms is usually detectable only at advanced stages, early detection of preclinical alterations and pathological biomarkers within the core brain regions is therefore essential for the diagnosis and initiation of interventional strategies to alleviate the neurodegenerative process. Promisingly, with the implementation of specific radiotracers, position emission tomography (PET) or single-photon emission computed tomography (SPECT) can accurately estimate striatal dopamine depletion in vivo, which is regarded as one of the diagnostic imaging markers for PD.

Recent neuroimaging studies have emphasized that white matter changes might also underlie the neurodegenerative processes in PD, despite the primary role of dopaminergic dysfunction^2–4^. Disruptions of microstructural integrity in the SN and in olfactory fibre tracts have been reported by using diffusion tensor imaging (DTI), a promising magnetic resonance imaging (MRI) analysis form of diffusion weighted imaging (DWI) for quantifying structural changes at the cellular level^5,6^. Previous studies have demonstrated that motor deficits in PD were correlated with dopamine synthesis capacity assessed by PET^7^ or aberrant diffusion metrics detected by DTI^8^ and deduced a probable dopaminergic effect on white matter integrity. Indeed, the combination of PET and DTI would crucially contribute to a better understanding of the association between these two aspects of the pathophysiological alterations in PD. However, whether microstructural integrity in subcortical regions and nigrostriatal projections have an effect on dopamine concentrations and consequently induce motor deficits remains unconfirmed based on sensitive neuroimaging biomarkers, given that confounding pharmacological treatments, asymmetric effects, and methodological differences in PD-related studies^9–11^.

Advances in neuroimaging have provided further insight into the pathophysiology of PD. Being capable of simultaneous acquisitions, a hybrid PET-MRI modality could provide highly accurate assessments of metabolic, functional, and structural information under the same physiological state^12^. Choi et al.^13^ initially applied hybrid PET-MRI to evaluate the concordance between grey matter changes and striatal dopaminergic degeneration in PD patients. Nonetheless, how early dopaminergic dysfunction correlates with microstructural disorganization of key subcortical regions and nigrostriatal pathways under the same spatial and temporal conditions has not yet been reported. Therefore, we conducted a hybrid PET-MRI study with a cohort of de novo, drug-naïve, and non-demented PD patients in the early stages and comprehensively investigated the early disruptions in dopamine concentrations, integrity in subcortical regions (including the SN, components of the basal ganglia), and microstructure of nigrostriatal pathways (fibre tracts from the SN to the striatum). The correlations among reduced striatal dopamine uptake, aberrant DTI metrics, and motor dysfunction were further explored systemically, while taking asymmetric effects into consideration. Importantly, mediation analysis was performed to examine whether dopaminergic dysfunction mediate the influence of microstructural nigrostriatal damage on movement disorders in the early stages.

## Results

### Demographic and clinical characteristics

A summary of the demographic and clinical data is given in Table 1. The PD and healthy controls (HC) were matched for age, sex, education, and Montreal Cognitive Assessment (MoCA) scores. These newly diagnosed PD patients were in the early stages with a Hoehn and Yahr (H-Y) stage of 1.28 ± 0.45 and a disease course of 17.44 ± 5.19 months.

**Table 1:** Demographic and clinical characteristics of the participants included in the study.

	HC (*n* = 24)	PD (*n* = 25)	*T*	*P*	*ES*
Age	64.00 ± 10.44	65.96 ± 14.77	−0.53	0.59	0.15
Gender (M/F)	8/16	7/18	0.26	0.61	0.15
Education (years)	10.58 ± 3.51	10.80 ± 3.71	−0.21	0.84	0.060
Disease course (months)	–	17.44 ± 5.19	–	–	–
UPDRS-III	–	26.88 ± 8.73	–	–	–
H-Y stage	–	1.28 ± 0.45	–	–	–
MoCA	28.67 ± 1.34	28.24 ± 1.27	1.45	0.26	0.35
Asymmetric Onset (L/R)	–	14/11	–	–	–
Asymmetric index (L/R)	–	−0.74 ± 0.24/0.76 ± 0.27	–	–	–

Data was represented as mean ± standard deviation.

Abbreviations: *HC* healthy control, *PD* Parkinson’s disease, *ES* effective size, *M* male, *F* female, *UPDRS* Unified Parkinson’s Disease Rating Scale, *H-Y* Hoehn-Yahr, *MoCA* Montreal Cognitive Assessment, *L* left, *R* right.

### Striatal standardized uptake value ratio (SUVR) differences and correlations

**Fig. 1:**
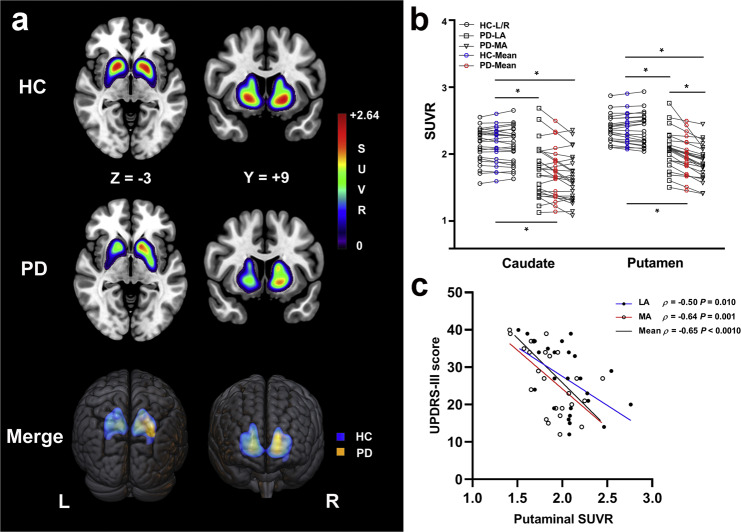
^18^F-DOPA SUVR parametric images and scatter plots. **a** Images of HC subject (M, age 62 years, MoCA score 30) and PD patient (F, age 63 years, MoCA score 29, UPDRS-III score 12, H-Y stage 1). These two parametric images were merged for the demonstration of the extent of striatal SUVR. The colorbar represents the value of SUVR. **b** Scatter plots of caudal and putaminal SUVR in HC subjects and PD patients. The striatal SUVR was from left and right side in HC subjects, and MA and LA side of symptom onset in PD patients separately. **c** Correlation between putaminal SUVR (separately for LA, MA, and mean; *X*-axis) and scores of UPDRS-III (*Y*-axis) in PD group. * indicates significant difference (*P* < 0.05) between groups. Solid line shows significant correlation. Abbreviations: ^18^F-DOPA ^18^F-fluorodopa, SUVR standard uptake value ratio, M male, F female, HC healthy controls, PD Parkinson’s disease, UPDRS-III Unified Parkinson’s Disease Rating Scale III, MoCA Montreal Cognitive Assessment, H-Y Hoehn and Yahr, L left, R right, LA less affected, MA more affected.

A typical example of a striatal ^18^F-fluorodopa (^18^F-DOPA) uptake image and scatter plots for the HC and PD groups are provided in Fig. 1a. Mean ^18^F-DOPA SUVR values in the putamen and caudate were markedly reduced in the PD patients (Putamen, *t* = 6.01, *P* < 0.001; Caudate, *t* = 4.81, *P* < 0.001) compared with the HCs, as well as on the less affected (LA) side and more affected (MA) side (Putamen, *F* = 24.29, *P* < 0.001, LA, *t* = 4.49, *P* < 0.001, MA, *t* = 6.88, *P* < 0.001; Caudate, *F* = 13.95, *P* < 0.001, LA, *t* = 3.93, *P* < 0.001, MA, *t* = 5.04, *P* < 0.001) (Fig. 1b). The putaminal SUVR on the MA side was significantly lower than that on the LA side (*t* = 2.20, *P* = 0.031), whereas no significant caudal SUVR differences were found between the MA side and LA side (*t* = 1.13, *P* = 0.27) (Fig. 1b). In the putamen, the bilateral and mean SUVR were all negatively correlated with Unified Parkinson’s Disease Rating Scale III (UPDRS-III) scores (LA, *ρ* = −0.50, *P* = 0.010; MA, *ρ* = −0.64, *P* = 0.001; Mean, *ρ* = −0.65, *P* < 0.001) (Fig. 1c). We failed to observe significant correlations between SUVR values in the caudate and UPDRS-III scores (LA, *P* = 0.20; MA, *P* = 0.73; Mean, *P* = 0.46).

### DTI alterations in subcortical regions and correlations

**Fig. 2:**
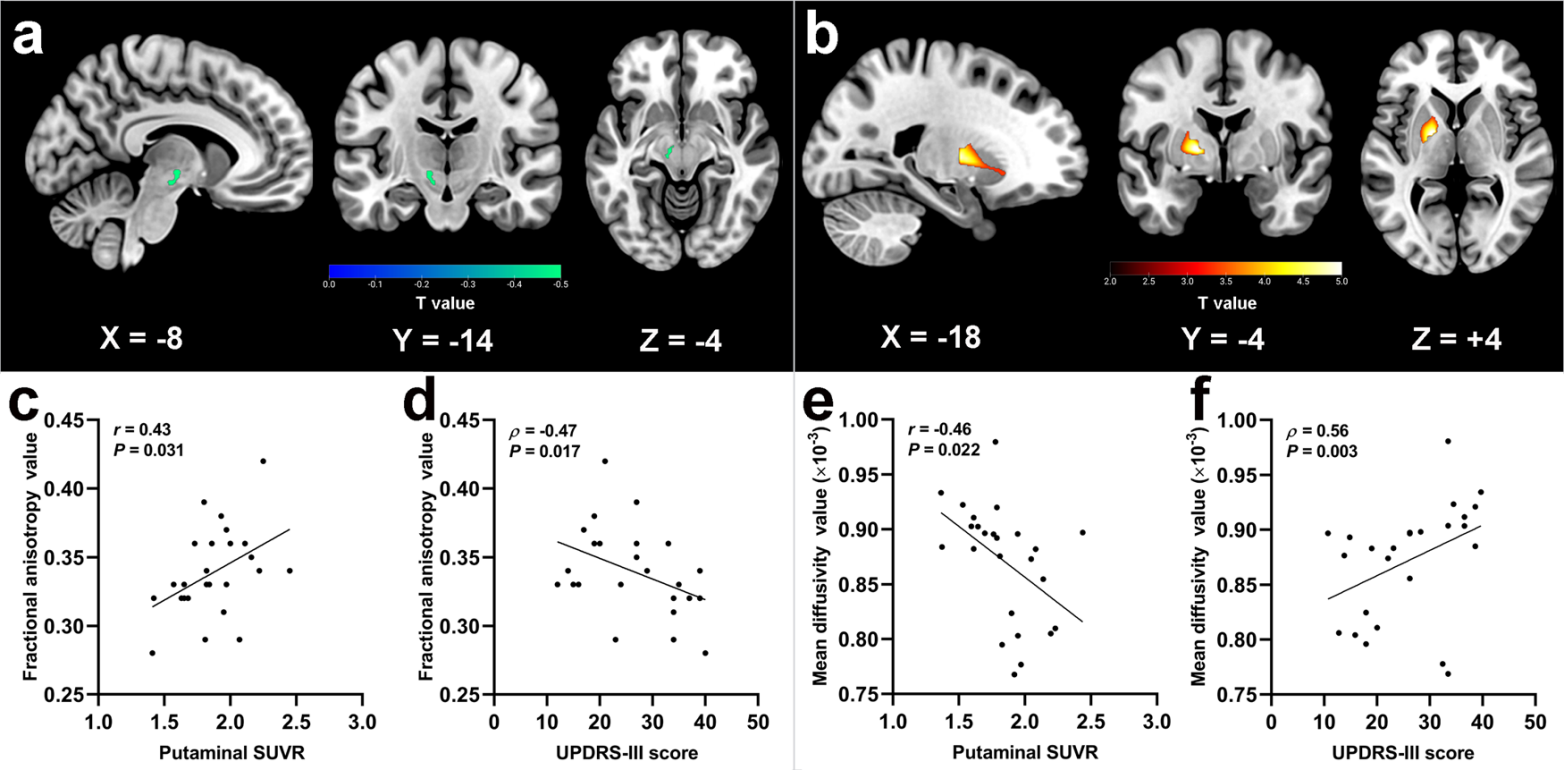
The significantly different brain regions of FA and MD maps between groups, and scatter plots of correlations in PD group. Brain regions in MA side with significantly decreased FA (**a**) and increased MD (**b**) in PD group as compared with HC group. **c** Correlation between FA values of substania nigra (*X*-axis) and putaminal SUVR (*Y*-axis). **d** Correlation between FA values of substania nigra (*X*-axis) and UPDRS-III scores (*Y*-axis). **e** Correlation between MD values of putamen (*X*-axis) and putaminal SUVR (*Y*-axis). **f** Correlation between MD values of putamen (*X*-axis) and UPDRS-III scores (*Y*-axis). The voxel-based analysis findings correspond to a voxel-wise height threshold of *P* < 0.001 (uncorrected) combined with a cluster-level extent threshold of *P* < 0.05 (corrected for multiple comparisons using the FWE rate). The cold colorbar represents the significantly decreased FA in the patients with PD. The warm colorbar indicates the significantly increased MD in the patients with PD. Solid line in the scatter plots shows significant correlation (*P* < 0.05). Abbreviations: FA fractional anisotropy, MD mean diffusivity, SUVR standard uptake value ratio, HC healthy control, PD Parkinson’s disease, UPDRS-III Unified Parkinson’s Disease Rating Scale III, MA more affected.

The Voxel-based analysis (VBA) analysis revealed no surviving significant clusters for the fractional anisotropy (FA) or mean diffusivity (MD) maps between the PD and HC groups. However, significantly decreased FA in the SN and increased MD in the putamen were observed in PD patients on the MA side compared with HC subjects (Fig. 2a, b). The brain regions with significant group differences in FA and MD are shown in Table 2. The FA values in the significant cluster showed a positive correlation with putaminal SUVR values and a negative correlation with UPDRS-III scores (*r* = 0.43, *P* = 0.031; *ρ* = −0.47, *P* = 0.017) (Fig. 2c, d). We also found that the increased MD values in the significant cluster were correlated with putaminal dopamine reduction and motor impairment (*r* = −0.46, *P* = 0.022; *ρ* = 0.56*, P* = 0.003) (Fig. 2e, f).

**Table 2:** Descriptions of brain regions with significant group differences in fractional anisotropy and mean diffusivity.

DTI	Brain regions (AAL)	Peak MNI coordinates (mm)			Peak *T* value	Cluster size (mm^3^)
		*X*	*Y*	*Z*		
Fractional anisotropy	PD < HC					
	Left substania nigra	−8	−14	−4	−6.14	39
Mean diffusivity	PD > HC					
	Left putamen	−18	−4	4	5.73	265

These findings correspond to a voxel-wise height threshold of *P* < 0.001 (uncorrected) combined with a cluster-level extent threshold of *P* < 0.05 (corrected for multiple comparisons using the FWE rate).

Abbreviations: DTI diffusion tensor imaging, AAL automated anatomical labelling, MNI Montreal Neurological Institute, HC healthy control, PD Parkinson’s disease.

### Nigrostriatal fibre damage and correlations

**Fig. 3:**
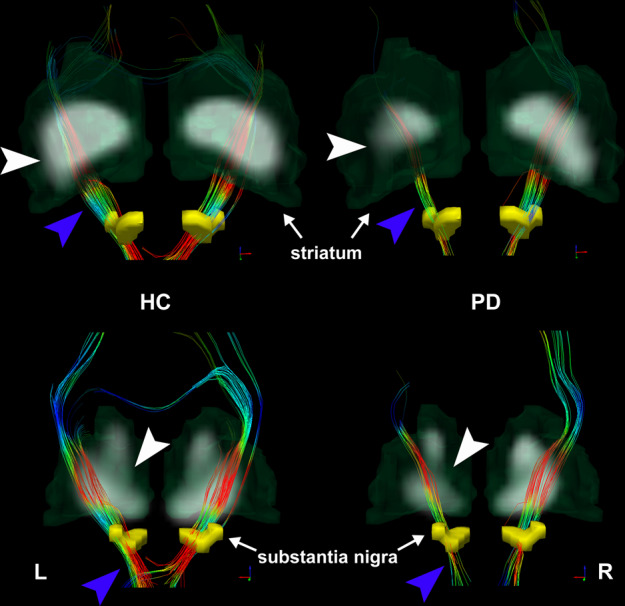
Deterministic tractography images of reconstructed nigrostriatal fibre tracts (projected from substantia nigra to striatum) coregistering with ^18^F-DOPA SUVR parametric images. With different projections, impaired nigrostriatal fibre tracts (blue arrowhead) were distinct in accordance with reduced striatal SUVR (white arrowhead). Colour-encoded orientations: green for anterior–posterior, red for transverse, and blue for superior–inferior directions. Abbreviations: ^18^F-DOPA ^18^F-fluorodopa, SUVR standard uptake value ratio, HC healthy control, PD Parkinson’s disease, L left, R right.

**Fig. 4:**
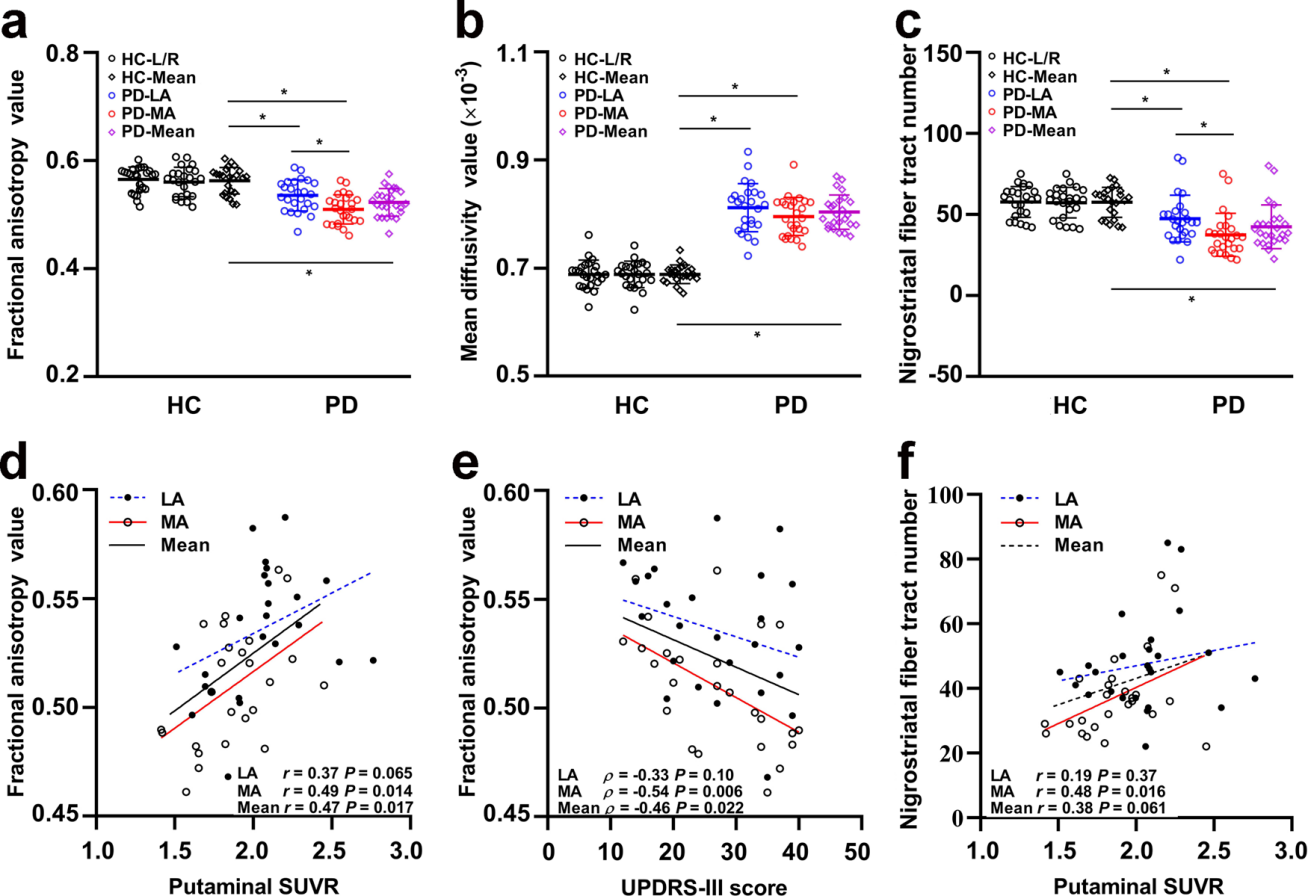
Scatter plots of group differences in deterministic tractography, and correlations for PD subjects. Group differences of bilateral FA (**a**), MD (**b**), and TN (**c**) in HC subjects and PD patients, separately for MA and LA side of symptom onset in PD patients. **d** Correlation between the corresponded SUVR of putamen UPDRS-III (*X*-axis) and the FA values (*Y*-axis). **e** Correlation between the scores of UPDRS-III (*X*-axis) and the FA values (*Y*-axis). **f** Correlation between the TN values (*X*-axis) and the corresponded SUVR of putamen (*Y*-axis). * indicates significant difference (*P* < 0.05) between groups. Solid line shows significant correlation (*P* < 0.05). Dotted line indicates none significant correlation (*P* > 0.05). Abbreviations: FA fractional anisotropy, MD mean diffusivity, TN tracts numbers, LA less affected, MA more affected, SUVR standard uptake value ratio, HC healthy control, PD Parkinson’s disease, UPDRS-III Unified Parkinson’s Disease Rating Scale III, L left, R right.

The nigrostriatal fibre tracts were well described by deterministic tractography. The impaired nigrostriatal fibre tracts were distinct in accordance with reduced striatal SUVR, as the example in Fig. 3 shows. The scatter plots for the group differences and correlations are shown in Fig. 4. The mean FA, MD, and tract numbers (TN) of nigrostriatal fibres were all significantly decreased in PD patients with respect to the HC subjects (FA, *t* = 5.58, *P* < 0.001; MD, *t* = −15.78, *P* < 0.001; TN, *t* = 4.12, *P* < 0.001), as well as on the LA side and MA side (FA, *F* = 22.87, *P* < 0.001, *t* = 3.50, *P* = 0.001, *t* = 6.76, *P* < 0.001; MD, *F* = 82.66, *P* < 0.001, *t* = 11.88, *P* < 0.001, *t* = 10.30, *P* < 0.001; TN, *F* = 10.88, *P* < 0.001, *t* = 2.37, *P* = 0.020, *t* = 4.67, *P* < 0.001) (Fig. 4a–c). An asymmetric effect was observed in the FA and TN of nigrostriatal fibres, with lower values on the MA side than on the LA side (FA, *t* = 3.30, *P* = 0.002; TN, *t* = 2.32, *P* = 0.023) (Fig. 4a, c). There were no significant differences in MD found between the LA side and MA side (*t* = 1.59, *P* = 0.12) (Fig. 4b). For mean and MA side measurements, positive correlations between FA values in nigrostriatal projections and SUVR values in the putamen all survived multiple comparison correction (MA, *r* = 0.49, *P* = 0.014; Mean, *r* = 0.47, *P* = 0.017) (Fig. 4d), while negative correlations with UPDRS-III scores were observed (MA, *ρ* = −0.54, *P* = 0.006; Mean, *ρ* = −0.46, *P* = 0.022) (Fig. 4e). However, there were no significant correlations observed on LA side (SUVR, *P* = 0.065; UPDRS-III, *P* = 0.10). The reduced TN on the MA side was significantly correlated with decreased putaminal ^18^F-DOPA SUVR values (*r* = 0.48, *P* = 0.016) (Fig. 4f), although none of the significant correlations were survived on mean (*P* = 0.061) and LA side (*P* = 0.37). We did not find significant correlations between MD values (LA, *P* = 0.65; MA, *P* = 0.94; Mean, *P* = 0.89) and TN (LA, *P* = 0.27; MA, *P* = 0.12; Mean, *P* = 0.068) of nigrostriatal projections and UPDRS-III scores, nor survived correlations between MD values and putaminal SUVR values (LA, *P* = 0.75; MA, *P* = 0.76; Mean, *P* = 0.94).

### Mediation effect of dopaminergic dysfunction

**Fig. 5:**
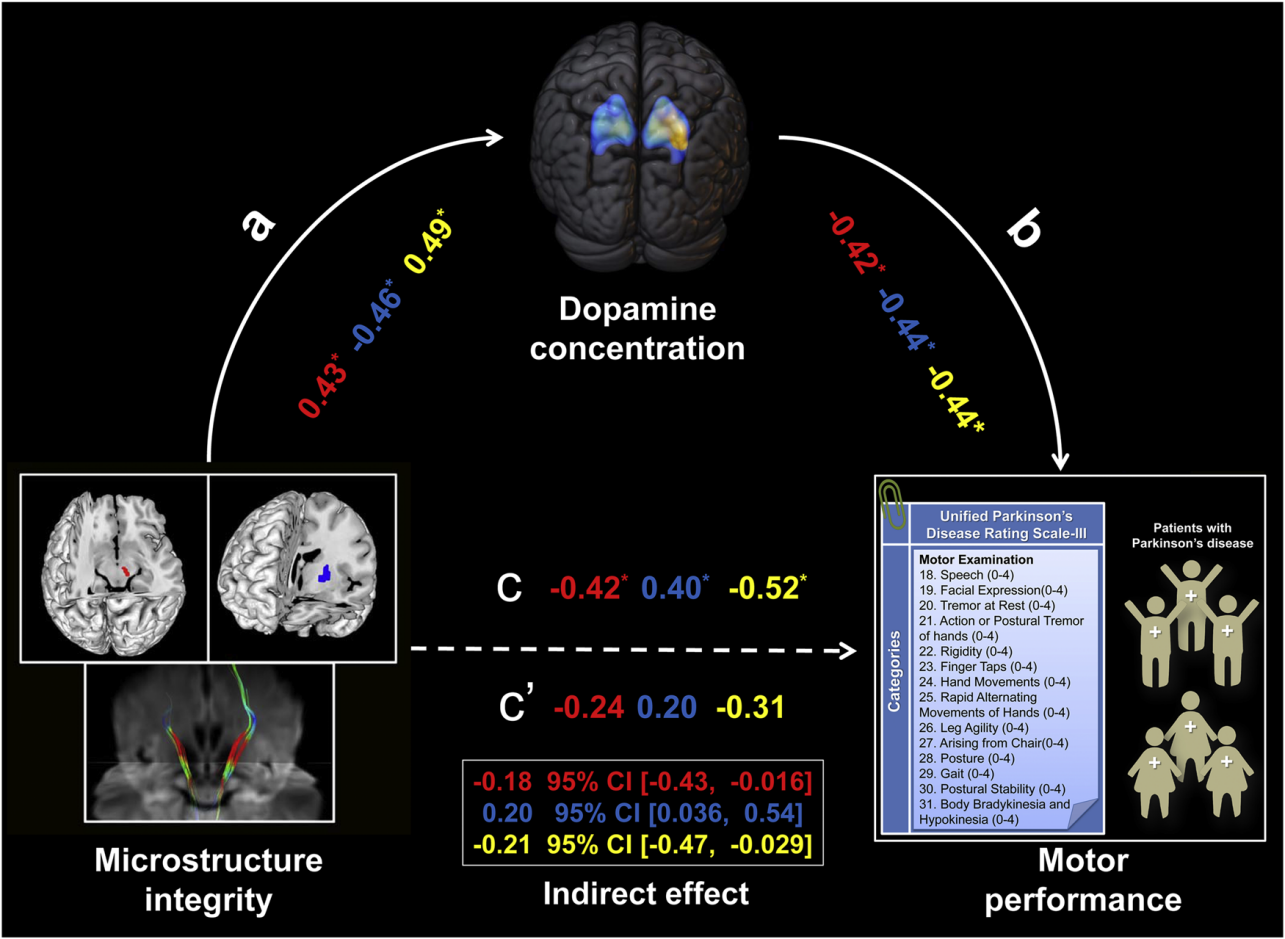
The mediation effect of dopaminergic function on the more affected side of PD. Dopamine concentration (putaminal SUVR) significantly mediated the relationship between microstructure integrity (FA in SN, red; MD in putamen, blue; FA of nigrostriatal projection, yellow) and motor performance (UPDRS-III scores). a, b, c, and c’ indicate path coefficients; * indicates significant difference (*P* < 0.05). Abbreviations: PD Parkinson’s disease, SUVR standard uptake value ratio, FA fractional anisotropy, MD mean diffusivity, UPDRS-III Unified Parkinson’s Disease Rating Scale III, CI confidence interval.

The mediation analysis and the investigated variables are described in Fig. 5. On the MA side, the significant associations between DTI metrics (nigral FA, putaminal MD, and FA of nigrostriatal projection) and UPDRS-III scores were significantly mediated by putaminal SUVR, respectively (indirect effect: −0.18, 95% CI: −0.43, −0.016; indirect effect: 0.20, 95% CI: 0.036, 0.54; indirect effect: −0.21, 95% CI: −0.47, −0.029). For the mediation pathway with dopamine concertation as independent variable, we failed to find any statistically significant results (all *P* > 0.05).

## Discussion

In the current study, we implemented hybrid PET-MRI, which is superior for the synchronous detection of different aspects of brain alterations, and systemically investigated the early molecular changes and microstructural degradation within the dopaminergic system in PD patients and obtained several principal findings. In comparison to HC subjects, PD patients demonstrated early dopamine depletion in putamen and microstructural damage in the SN, striatum, and nigrostriatal fibre tracts, which were significantly correlated with clinical motor deficits. Meanwhile, the reduced dopamine synthesis capacity and microstructural integrity were pronounced on the MA side, thus indicating that asymmetric effects should be considered a major impact on the heterogeneity in PD. Mediation analysis further indicated that dopamine depletion was a significant mediator, providing supporting neuroimaging evidence that microstructural integrity in the nigrostriatal system modulating motor performance might be through dopaminergic dysfunction. Further studies are warranted to validate the effectiveness of this technology for assisting in monitoring PD progression and evaluating the effects of pharmacological treatments.

By using a radiotracer probe with selective affinity in the striatum, PET can provide dopamine-related information on the central pathology of PD^14^. Our ^18^F-DOPA PET findings were in accordance with previous reports^15,16^, consistently demonstrating that dopamine depletion was more selective in the putamen of the MA side and was correlated with PD disease severity. Emerging evidence in recent studies^17,18^ has confirmed that hybrid PET-MRI is superior to PET-CT with improved spatial resolution and higher diagnostic accuracy. Furthermore, this approach acquires both PET and MRI synchronously, which is more efficient for multimodal studies^19,20^ with respect to combined imaging modalities. A previous study^13^ reported only the relationship between cortical changes and striatal dopamine loss in medicated PD patients due to a lack of HC participants. Our current study, focusing on unmedicated PD patients in the early stages, validated hybrid PET-MRI for the investigation of significant neurochemical alterations, microstructural damage, and correlations among the related pathological processes.

Neuronal impairments in subcortical grey matter are of great concern in the pathology of PD. Although diffusion metrics derived from DTI are generally regarded as a proxy for microstructural degeneration, altered FA or MD in the subcortical grey matter remains a controversial issue due to heterogeneity among PD patients. By using the VBA approach, we failed to find any significant regions in DTI maps (FA and MD) when comparing PD and HC subjects. However, significant differences survived after controlling the asymmetric effect by flipping the MA side of individuals to the same side. In agreement with the prevailing perspective that nigral diffusion alterations represent the main consensus^21–23^, a significantly decreased FA in the SN was also detected in the present study, along with a correlation with disease severity, despite lateralization. Meanwhile, our findings of a significantly increased MD in the putamen on the MA side, as well as correlations between MD values and UPDRS-III scores, were in line with previous literature^22,24^, presumably indicating pathological alterations in tissue microstructure related to motor deficits in the early stages of PD. Further studies are still warranted for the determination of definite microstructural alteration within nigrostriatal pathway in subcortical regions.

Evidence from neuropathological studies has demonstrated that microstructural damage in PD proceeds along major fibre pathways^25^; thus, we further attempted to investigate the integrity and numbers of nigrostriatal fibre tracts. Zhang et al.^9^ performed deterministic tractography by using ROIs that consisted of the SN, putamen and globus pallidus, which were drawn manually. In order to be more accurate and decrease the inter-rater variability, the present study tracked projections from the SN to the putamen and caudate, the primary regions innervated by the nigrostriatal pathway^26^, with automated ROIs and analyzed correlations with simultaneously determined striatal dopamine concentrations. As a pronounced finding in our study, the reduced FA and numbers in the nigrostriatal tract were significantly different between groups and between unilateral sides, providing an informative basis for the differential diagnoses and asymmetry confirmation. In particular, the inverse correlation between FA values and UPDRS-III scores at the group level and on the MA side further reinforced that the degeneration of nigrostriatal pathways is potentially relevant to disease severity.

Increasing attention has been focused on the associations between dopaminergic impairments and integrity of the basal ganglia. Scherfler et al.^5^ conducted an ROI-based study in PD patients and found a correlation between nigral MD changes and putaminal ^18^F-DOPA SUVR values. Although our results also supported this notion, we observed significantly decreased FA rather than increased MD in the SN by using the VBA approach, which was in accordance with the existing literature^6,27^. By using PET-DTI combined measurements, Kawaguchi et al.^28^ reported a negative correlation between dopamine synthesis capacity and MD in the striatum, which was partially supported by our VBA results, and suggested that dopaminergic function may be related to the density of dopaminergic neuronal fibres since MD reflects the density of widely spreading axonal terminals. However, this assumed relationship has not been directly confirmed in previous studies^23^ due to a lack of nigrostriatal fibre tracing and striatal dopamine concentration measurements. In our study, on the basis of supporting evidence derived from advanced hybrid PET-MRI, we linked the integrity of nigrostriatal fibres to dopaminergic function in the early stages of PD without intervention. Most importantly, the mediation effect of putaminal presynaptic dopaminergic dysfunction in this study strengthened the insight into the underlying aetiology, suggesting that PD evolves with an asymmetric pattern of microstructural degradation (at the level of the SN, striatum, and neural fibres in the nigrostriatal pathway), which drives motor deficits potentially through dopaminergic dysfunction.

Some limitations of the current study should be considered. First, the sample size in this demonstrative study was relatively small for the statistical analysis, and a larger-scale cohort including comprehensive motor and cognitive assessments is required in future studies. Second, we focused on the initial disruptions in the nigrostriatal system in the early stages of PD, and the examination of alterations in other related fibre tracts and cortical regions during disease progression is warranted for hybrid PET-MRI research in the future. Third, more PET tracers specific to non-dopaminergic systems (e.g. serotonergic system, cholinergic system, noradrenergic system) and other functional MRI analyses will be important issues for hybrid PET-MRI studies to better demonstrate the underlying mechanisms of PD heterogeneity. Finally, although several outcomes were obtained by using the VBA approach and automated ROIs to minimize variability, more sophisticated processing methods and advanced diffusion sequences (e.g. free-water diffusion imaging) that beyond FA/MD metrics and specific to nigrostriatal pathway are still essential for validation.

In conclusion, our demonstrative hybrid PET-MRI study first demonstrated detectable PET and DTI abnormalities accompanied by asymmetric effects within the dopaminergic system while further characterizing the close association and mediating pathway among microstructural disorganization, molecular degeneration, and motor dysfunction in the early stages of PD. Our supporting neuroimaging evidences validated that DTI and PET metrics acquired simultaneously by hybrid PET-MRI could contribute to the investigation of the underlying pathology involving dopaminergic deficits in PD individuals.

## Methods

### Participants

From August 2019 to June 2020, a cohort of 25 (7 males and 18 females) newly diagnosed, untreated, and non-demented PD patients who visited the Movement Disorders outpatient clinic and received hybrid PET-MRI scans were consecutively enroled in this study. We also recruited 24 (8 males and 16 females) HC matched for age, sex, and years of education. All participants were right-handed. Two experienced neurologists (Youyong Tian, with 30 years of experience, and Hongdong Zhao, with 33 years of experience) who administered a structured interview to subjects and their informants performed the inclusion and exclusion assessments. This study was performed with approval from the local institutional review board of Nanjing Medical University, and written informed consent was obtained 24 h prior to the examinations.

For each patient, the PD-related clinical features were evaluated with the following assessment tools: (1) disease severity was scored by using the UPDRS-III; (2) disease stage was classified according to the H-Y rating scale; and (3) clinical laterality was determined by the asymmetry index (AI)^29^, which was calculated by UPDRS-III scores (items 20–26), and matched the initial side of motor disability. The global cognitive function of all subjects was scored using the MoCA. With reference to clinical laterality, the hemisphere contralateral to the more affected body side was defined as the MA side, and the other hemisphere was defined as the LA side^30^.

The following inclusion criteria were used for PD patients: (1) diagnosed with United Kingdom Parkinson’s Disease Society Brain Bank clinical diagnostic criteria^31^; (2) evidence of dopamine deficiency on ^18^F-DOPA PET-MRI imaging; (3) disease duration (from first symptom) not more than 2 years and H-Y stage not higher than II; and (4) no history of any antiparkinsonian treatments. The exclusion criteria for all subjects were as follows: (1) family history of PD, secondary parkinsonism, or parkinsonism syndrome; (2) any neuropsychiatric disorder, such as Alzheimer’s disease, epilepsy, seizures or any psychiatric disease; (3) any other disease related to the nervous system, including central nervous system infection, cerebrovascular disorders, neurological surgery, major head injury, brain tumours, diabetes, or a history of alcohol and/or drug abuse; (4) treatments with psychotropic agents or anticholinergic drugs; (5) cognitive impairment with MoCA score <26; 6) any contraindication for MRI, including claustrophobia, ferromagnetic foreign bodies, and electronic implants; (7) severe handicaps (e.g. vision or hearing loss) that would interfere with neuropsychological assessments or study procedures; and (8) excessive head motion found in data preprocessing.

### Data acquisition

All subjects fasted for a minimum of 6 h and received blood glucose measurements upon arrival at the PET centre. Administration of radiotracer would proceed only if glucose levels were below 120 mg/dl. In all cases, carbidopa (2.5 mg/kg) was orally administered 1 h prior to intravenous injection of ^18^F-DOPA (200 MBq, synthesized by the PET centre of our hospital; radiochemical purity ≥95%). Static PET-MRI acquisition was conducted 90 min following the injection.

PET-MRI data were simultaneously acquired using a 3.0-tesla hybrid PET-MRI scanner (uPMR 790, United Imaging Healthcare, Shanghai, China) with a commercial 32-channel head coil. The participants wore headphones and were instructed to lie in a supine position while staying awake with their eyes closed and to avoid thinking of anything in particular during the scan.

A single-bed-position, 18-minute PET reconstruction through the head was generated using the list mode data set. The PET reconstruction parameters were 256 × 256 matrix, 300 × 300 mm^2^ field of view (FOV), ordered subset expectation maximization reconstruction algorithm with point spread function and time-of-flight modelling, 28 subsets, 4 iterations, and 3-mm post filter. Atlas-based attenuation correction was used to generate brain PET images. The standardized uptake value (SUV) was calculated by using the following formula: SUV = *c*_dc_/(*d*_i_/*w*), where *c*_dc_ is the decay-corrected tracer tissue concentration (in Bq/g), *d*_i_ is the injected dose (in Bq), and *w* is the patient’s body weight (in g).

The following MRI sequences were sequentially acquired during the PET scan: (a) transverse echo planar imaging sequence-based DTI with 32 gradient directions: *b* value = 0, 1000 s/mm^2^; repetition time/echo time (TR/TE) = 4663/78 ms; FOV = 230 × 250 mm^2^; slice thickness = 4 mm; matrix = 118 × 128; flip angle (FA) = 90°; bandwidth (BW) = 1630 Hz; acceleration factor = 2; total time = 8 min 20 sec. (b) Three-dimensional T1-weighted spoiled gradient-recalled echo sequence (3D T1-GRE): TR/TE = 7.86/3.2 ms; inversion time (TI) = 810 ms; FOV = 232 × 256 mm^2^; slice thickness = 0.71 mm; matrix = 348 × 384; FA = 10°; BW = 250 Hz; total time = 5 min 30 sec.

### Grey matter volume calculation

The grey matter volume (GMV) of each voxel was calculated and used as one of covariates for the correction of atrophy or partial volume effects. SPM12 was used for GMV calculation. Segmentation was performed for the T1 anatomic maps, by dividing white matter, grey matter (GM), and cerebrospinal fluid with the standard uniform segmentation model. The GM concentration maps were initial affine registered to MNI space, nonlinear deformation of GM concentration maps were then processed using the diffeomorphic anatomical registration through exponentiated Lie algebra technique, and were resampled to a voxel size of 1.5 × 1.5 × 1.5 mm^3^. The GMV of each voxel was obtained by multiplying the GM concentration graph by the nonlinear determinant obtained by the spatial normalization step. Then, the GMV maps were smoothed with a 6 × 6 × 6 mm^3^ FWHM Gaussian kernel. Finally, the mean GMV of each individual were extracted.

### PET/MRI data preprocessing and analysis

The PET data were preprocessed using Statistical Parametric Mapping (SPM, version 12, https://www.fil.ion.ucl.ac.uk/spm) running in MATLAB R2016b (MathWorks Inc., Natick, MA, USA). The procedures were performed as follows^10^: each participant’s PET image was coregistered to their structural T1 images, and individual structural images were normalized to Montreal Neurological Institute (MNI) space; spatial transforms were concatenated to bring the PET image to the MNI template, with resampling to a 2 mm × 2 mm × 2 mm voxel size. Finally, the spatially normalized PET images were then smoothed using an 8 mm full-width at half-maximum (FWHM) Gaussian kernel. The bilateral putamen, caudate nucleus, and calcarine cortex were selected as seed regions of interest (ROIs) by the automated anatomical labelling atlas^32^. ^18^F-DOPA SUVR values for these ROIs were extracted. The striatal SUVR was calculated as the mean uptake value of the striatal subregion/mean uptake value of the calcarine cortex^33^.

The DTI data were preprocessed with FMRIB’s Software Library (FSL, version 4.1.8; Oxford Centre for Functional MRI of the Brain, Oxford, UK) software (http://www.fmrib.ox.ac.uk/fsl). Eddy current distortions and head motion artefacts of each subject were corrected by using affine registration in FMRIB’s Diffusion Toolbox (FDT) 2.0^34^. A binary brain mask was created from the non-diffusion-weighted volume (b0) using the Brain Extraction Tool (BET v2.1) with a fractional threshold of 0.2 to non-brain structures before tensor fitting^35^. The FA and MD maps were generated for every voxel according to standard methods based on the diffusion tensors reconstructed with the DTIFIT program. Finally, the parametric images of each individual were spatially normalized to the standard MNI template, with resampling to a 2 mm × 2 mm × 2 mm voxel size, using FMRIB’s Linear Image Registration Tool (FLIRT).

VBA was conducted within the core subcortical grey matter. The processed FA and MD maps were analyzed using SPM 12. The spatially normalized FA and MD maps of each subject were smoothed to an FWHM Gaussian kernel of 8 mm. To explore the asymmetry effects, both FA and MD maps from the patients with left clinical laterality were flipped within whole brain, so that all predominantly affected sides appeared to be in the same hemisphere^36^. A two-sample *t*-test between the PD and HC groups was performed with sex, age, education, and individual mean GMV as covariates. The nonstationary cluster-level familywise error (FWE) method was used to correct for the results with a cluster-defining threshold of *P* < 0.001 and a corrected cluster significance of *P* < 0.05. A normalized mask obtained at a threshold of mean FA > 0.2 within subcortical grey matter was applied for the VBA.

Deterministic tractography of the nigrostriatal tract was conducted using TrackVis software (http://www.trackvis.org/)^9^. In brief, fibre tracking and normalization were performed with the Diffusion Toolkit program. To reduce variability in the manual segmentation, the bilateral SN and striatum (putamen, caudate, and globus pallidus) that were segmented by WFU_PickAtlas software (http://www.ansir.wfubmc.edu) were selected as target seed regions. A default length threshold of 10 mm and an angular threshold of 35 degrees were used to limit deviations from the main path. To avoid bias attributable to variable fibre lengths and terminations, measures of FA and MD were extracted every 1 mm from a 30-mm segment of the consistent portion of the streamlines. Mean values of FA and MD along the unilateral nigrostriatal tract segment as well as TN were recorded for further analysis.

### Statistics and reproducibility

Normality of the clinical and demographic data distributions was analyzed by the Kolmogorov–Smirnov test. Independent-samples *t*-tests (for age, striatal SUVR, FA/MD values of nigrostriatal tract, streamline numbers), a Mann–Whitney *U* test (for education and MoCA) and a chi-square (χ^2^) test (for sex) were applied to explore the differences between the PD and HC groups. Group difference (MA, LA, and HC) analyses of metrics for PET and tractography were performed using ANCOVA followed by post hoc Bonferroni tests. The effect size for demographic and clinical variables was also analyzed by using Cohen’s *d* (for age) or Phi coefficient (for sex, education, and MoCA).

The correlations between significant regions (FA or MD values) of VBA and clinical measurements (UPDRS-III, H-Y scale, MoCA, AI) were investigated separately using Spearman correlation analysis. The correlations between metrics for DTI and PET were investigated separately using Pearson correlation analysis. We further conducted mediation analysis by using PROCESS macro (http://www.processmacro.org/)^37^, for the purpose of determining whether the significant association between microstructure degradation (DTI metrics, independent variable) and motor symptoms (UPDRS-III scores, dependent variable) was mediated by dopaminergic dysfunction (putaminal SUVR, mediating variable). In case of bi-direction, we also tested another mediation pathway (dopaminergic dysfunction as independent variable and microstructure degradation as mediating variable). The analysis was based on 5000 bootstrap samples, and the significance of indirect effects was assessed by bootstrap 95% confidence interval (CI) that does not include zero. Sex, age, education, and GMV were considered as covariates^38^.

The above statistical analyses were performed using SPSS software (version 25.0, SPSS Inc., Chicago, IL, United States). A *P* value < 0.05 was defined as statistically significant.

### Reporting summary

Further information on research design is available in the Nature Research Reporting Summary linked to this article.

## Supplementary information

Description of Additional Supplementary Files

Supplementary Data 1

Reporting Summary

featured image

Language editting certificate

## Data Availability

The data supporting this study are available within the paper and Supplementary Data 1. Any additional data relating to the study are available from the corresponding author on reasonable request.
