# Supplementary material for: Hybrid PET-MRI for early detection of dopaminergic dysfunction and microstructural degradation involved in Parkinson’s disease
Source: Commun Biol. 2021 Oct 7;4:1162. doi: 10.1038/s42003-021-02705-x (PMC8497575; doi:10.1038/s42003-021-02705-x)
Supplement: Supplementary file 1 — Description of Additional Supplementary Files [file 42003_2021_2705_MOESM1_ESM.pdf]

Description of Additionaly Supplementary Files

File name: Supplementary data 1
Description: Source data behind the graphs in the paper.
