# Supplementary material for: Hybrid PET-MRI for early detection of dopaminergic dysfunction and microstructural degradation involved in Parkinson’s disease
Source: Commun Biol. 2021 Oct 7;4:1162. doi: 10.1038/s42003-021-02705-x (PMC8497575; doi:10.1038/s42003-021-02705-x)
Supplement: Supplementary file 3 — Reporting Summary [file 42003_2021_2705_MOESM3_ESM.pdf]

Corresponding author(s): DBPR COMMSBIO-21-1116B

Last updated by author(s): Sep 9, 2021

Reporting Summary

Nature Portfolio wishes to improve the reproducibility of the work that we publish. This form provides structure for consistency and transparency in reporting. For further information on Nature Portfolio policies, see our Editorial Policies and the Editorial Policy Checklist.

Statistics

For all statistical analyses, confirm that the following items are present in the figure legend, table legend, main text, or Methods section.

n/a	Confirmed	
☐	☒	The exact sample size (n) for each experimental group/condition, given as a discrete number and unit of measurement
☐	☒	A statement on whether measurements were taken from distinct samples or whether the same sample was measured repeatedly
☐	☒	The statistical test(s) used AND whether they are one- or two-sided<br>*Only common tests should be described solely by name; describe more complex techniques in the Methods section.*
☐	☒	A description of all covariates tested
☐	☒	A description of any assumptions or corrections, such as tests of normality and adjustment for multiple comparisons
☐	☒	A full description of the statistical parameters including central tendency (e.g. means) or other basic estimates (e.g. regression coefficient) AND variation (e.g. standard deviation) or associated estimates of uncertainty (e.g. confidence intervals)
☐	☒	For null hypothesis testing, the test statistic (e.g. F, t, r) with confidence intervals, effect sizes, degrees of freedom and P value noted<br>*Give P values as exact values whenever suitable.*
☒	☐	For Bayesian analysis, information on the choice of priors and Markov chain Monte Carlo settings
☒	☐	For hierarchical and complex designs, identification of the appropriate level for tests and full reporting of outcomes
☐	☒	Estimates of effect sizes (e.g. Cohen's d, Pearson's r), indicating how they were calculated

Our web collection on statistics for biologists contains articles on many of the points above.

Software and code

Policy information about availability of computer code

Data collection	PET-MRI data were simultaneously acquired using a 3.0-tesla hybrid PET-MRI scanner (uPMR 790, United Imaging Healthcare, Shanghai, China) with a commercial 32-channel head coil.
Data analysis	Data were preprocessed using Statistical Parametric Mapping (SPM, version 12, https://www.fil.ion.ucl.ac.uk/spm) running in MATLAB R2016b (MathWorks Inc., Natick, MA, USA) or FMRIB's Software Library (FSL, version 4.1.8; Oxford Centre for Functional MRI of the Brain, Oxford, UK) software (http://www.fmrib.ox.ac.uk/fsl). Statistical analyses were performed using SPSS software (version 25.0, SPSS Inc., Chicago, IL, United States).

For manuscripts utilizing custom algorithms or software that are central to the research but not yet described in published literature, software must be made available to editors and reviewers. We strongly encourage code deposition in a community repository (e.g. GitHub). See the Nature Portfolio guidelines for submitting code & software for further information.

Data

Policy information about availability of data

All manuscripts must include a data availability statement. This statement should provide the following information, where applicable:

- Accession codes, unique identifiers, or web links for publicly available datasets
- A description of any restrictions on data availability
- For clinical datasets or third party data, please ensure that the statement adheres to our policy

The raw data supporting the conclusions of this article will be made available from the corresponding author upon reasonable request.

Field-specific reporting

Please select the one below that is the best fit for your research. If you are not sure, read the appropriate sections before making your selection.

[x] Life sciences [ ] Behavioural & social sciences [ ] Ecological, evolutionary & environmental sciences

For a reference copy of the document with all sections, see nature.com/documents/nr-reporting-summary-flat.pdf

Life sciences study design

All studies must disclose on these points even when the disclosure is negative.

Sample size	The number of the sample sizes are chosen according to previous literatures. However, we acknowledge that the sample size in this preliminary study was relatively small for the statistical analysis, which is one of the limitations in this study.
Data exclusions	The exclusion criteria for all subjects were as follows: 1) family history of PD, secondary parkinsonism, or parkinsonism syndrome; 2) any neuropsychiatric disorder, such as Alzheimer's disease, epilepsy, seizures or any psychiatric disease; 3) any other disease related to the nervous system, including central nervous system infection, cerebrovascular disorders, neurological surgery, major head injury, brain tumours, diabetes, or a history of alcohol and/or drug abuse; 4) treatments with psychotropic agents or anticholinergic drugs; 5) cognitive impairment with MoCA score < 26; 6) any contraindication for MRI, including claustrophobia, ferromagnetic foreign bodies, and electronic implants; 7) severe handicaps (e.g., vision or hearing loss) that would interfere with neuropsychological assessments or study procedures; and 8) excessive head motion found in data preprocessing.
Replication	The data analyses were repeated three times and had high reproducibility.
Randomization	The patients and healthy controls were randomly enrolled in to this study according to the inclusion and exclusion criteria. These participants were subsequently divided into patient group and healthy group according to their clinical assessment.
Blinding	The data of each participant were obtained from PET/ MRI scanning or structured scales without image scoring or other procedures that need blinding assessment.

Reporting for specific materials, systems and methods

We require information from authors about some types of materials, experimental systems and methods used in many studies. Here, indicate whether each material, system or method listed is relevant to your study. If you are not sure if a list item applies to your research, read the appropriate section before selecting a response.

Materials & experimental systems

n/a	Involved in the study	
[x]	[ ]	Antibodies
[x]	[ ]	Eukaryotic cell lines
[x]	[ ]	Palaeontology and archaeology
[x]	[ ]	Animals and other organisms
[ ]	[x]	Human research participants
[x]	[ ]	Clinical data
[x]	[ ]	Dual use research of concern

Methods

n/a	Involved in the study	
[x]	[ ]	ChIP-seq
[x]	[ ]	Flow cytometry
[ ]	[x]	MRI-based neuroimaging

Human research participants

Policy information about studies involving human research participants

Population characteristics	Twenty-five (7 males and 18 females) newly diagnosed, untreated and non-demented PD patients (age, 65.96±14.77 years) who visited the Movement Disorders outpatient clinic and received hybrid PET-MRI scans were consecutively enrolled in this study. We also recruited 24 (8 males and 16 females) HC (age, 64.00±10.44 years).
Recruitment	Newly diagnosed, untreated and non-demented PD patients who visited the Movement Disorders outpatient clinic and received hybrid PET-MRI scans were consecutively enrolled in this study.
Ethics oversight	This study was performed with approval from the local institutional review board of Nanjing First Hospital.

Note that full information on the approval of the study protocol must also be provided in the manuscript.

Magnetic resonance imaging

Experimental design

Design type	Resting state
Design specifications	Not applicable.
Behavioral performance measures	Not applicable.

Acquisition

Imaging type(s)	Diffusion
Field strength	3.0 T
Sequence & imaging parameters	(a) transverse echo planar imaging sequence-based DWI with 32 gradient directions: b value = 0, 1000 s/mm2; repetition time/echo time (TR/TE) = 4663/78 ms; FOV = 230 × 250 mm2; slice thickness = 4 mm; matrix = 118 × 128; flip angle (FA) = 90°; bandwidth (BW) = 1630 Hz; acceleration factor = 2; total time = 8 minutes 20 seconds.
Area of acquisition	Whole brain

Diffusion MRI ☒ Used ☐ Not used

Parameters: (a) transverse echo planar imaging sequence-based DWI with 32 gradient directions: b value = 0, 1000 s/mm2; repetition time/echo time (TR/TE) = 4663/78 ms; FOV = 230 × 250 mm2; slice thickness = 4 mm; matrix = 118 × 128; flip angle (FA) = 90°; bandwidth (BW) = 1630 Hz; acceleration factor = 2; total time = 8 minutes 20 seconds.

Preprocessing

Preprocessing software	Statistical Parametric Mapping (SPM, version 12, https://www.fil.ion.ucl.ac.uk/spm) running in MATLAB R2016b (MathWorks Inc., Natick, MA, USA) and FMRIB's Software Library (FSL, version 4.1.8; Oxford Centre for Functional MRI of the Brain, Oxford, UK) software (http://www.fmrib.ox.ac.uk/fsl).
Normalization	Each participant's PET image was coregistered to their structural T1 images, and individual structural images were normalized to Montreal Neurological Institute (MNI) space; spatial transforms were concatenated to bring the PET image to the MNI template, with resampling to a 2 mm× 2 mm × 2 mm voxel size. The DTI parametric images of each individual were spatially normalized to the standard MNI template, with resampling to a 2 mm× 2 mm × 2 mm voxel size.
Normalization template	MNI152
Noise and artifact removal	A binary brain mask was created from the non-diffusion-weighted volume (b0) with a fractional threshold of 0.2 to non-brain structures before tensor fitting.
Volume censoring	Not applicable.

Statistical modeling & inference

Model type and settings	A two-sample t-test between the PD and HC groups was performed with sex, age, education, and individual mean GMV as covariates.
Effect(s) tested	Not applicable.

Specify type of analysis: ☐ Whole brain ☐ ROI-based ☒ Both

Anatomical location(s): The bilateral substantia nigra and striatum (putamen, caudate and globus pallidus) were segmented according to Anatomical Automatic Labeling.

Statistic type for inference (See Eklund et al. 2016)	Voxel-based analysis
Correction	The nonstationary cluster-level familywise error (FWE) method was used to correct for the results with a cluster-defining threshold of P<0.001 and a corrected cluster significance of P<0.05.

Models & analysis

n/a	Involved in the study
☒	☐ Functional and/or effective connectivity
☒	☐ Graph analysis
☒	☐ Multivariate modeling or predictive analysis
