# Supplementary figures and images for: Hybrid PET-MRI for early detection of dopaminergic dysfunction and microstructural degradation involved in Parkinson’s disease
Source: Commun Biol. 2021 Oct 7;4:1162. doi: 10.1038/s42003-021-02705-x (PMC8497575; doi:10.1038/s42003-021-02705-x)

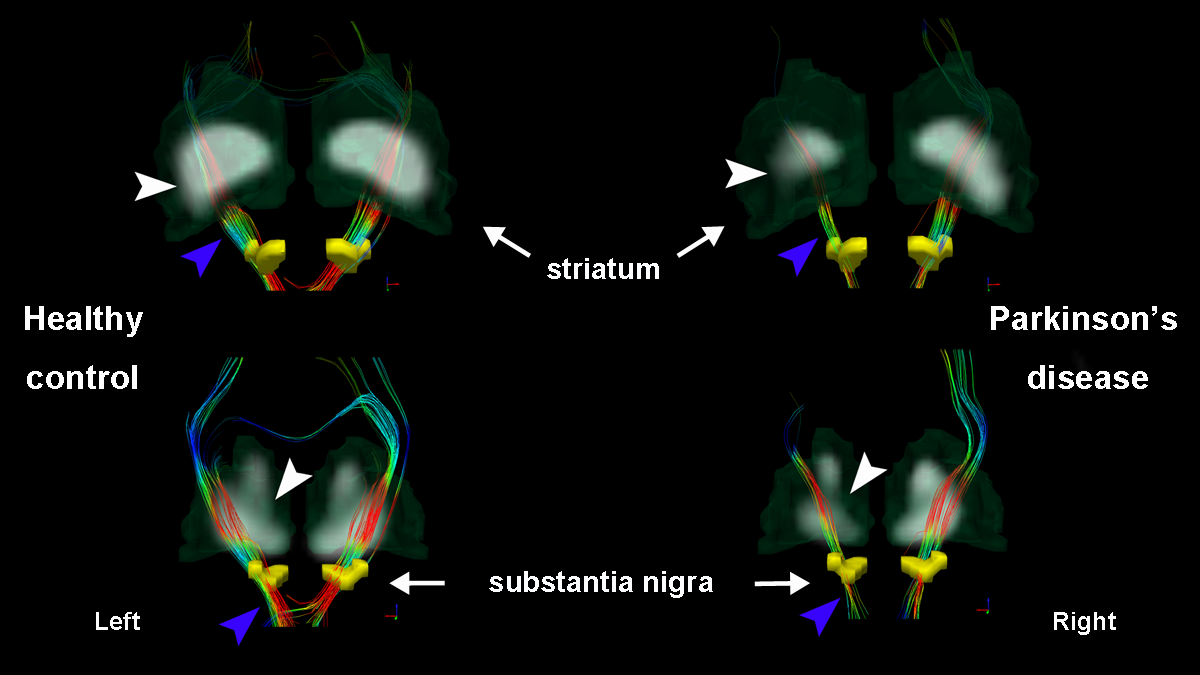

Supplement: Supplementary file 4 — featured image [file 42003_2021_2705_MOESM4_ESM.tif]
